# Effect of Coronavirus Disease 2019 Pandemic on Physical Activity in a Rural Area of Japan: The Masuda Study

**DOI:** 10.2188/jea.JE20200598

**Published:** 2021-03-05

**Authors:** Takashi Hisamatsu, Kaori Taniguchi, Mari Fukuda, Minako Kinuta, Noriko Nakahata, Hideyuki Kanda

**Affiliations:** 1 Department of Public Health, Okayama University Graduate School of Medicine, Dentistry and Pharmaceutical Sciences, Okayama, Japan; 2 Department of Environmental Medicine and Public Health, Izumo, Shimane University Faculty of Medicine, Shimane, Japan; 3 Department of Health and Nutrition, The University of Shimane Faculty of Nursing and Nutrition, Shimane, Japan

On March 11, 2020, the World Health Organization (WHO) declared coronavirus disease 2019 (COVID-19) to be a global pandemic.^1^ The Japanese government declared a nationwide state of emergency on April 16. Unlike other counties, the restrictions in Japan were not enforceable.^2^ Physical activity is an important determinant of health^3^ and is likely affected by social distancing measures. Daily step count is a proxy for physical activity, and its regional trends may be a proxy for adherence to social distancing, providing real-time insight to inform public policy decisions. We investigated the trends in step counts before and after WHO declaration of the COVID-19 pandemic.

The Masuda Study is an ongoing prospective cohort study to monitor daily trends in blood pressure, dietary nutrition, and physical activity using Internet of Things technologies among healthy community-dwelling individuals aged 20–74 years in Masuda, Shimane, Japan. From October 2018, 242 participants provided written informed consent and completed a survey of daily physical activity. This study was approved by the Institutional Review Board of Okayama University. We objectively measured daily step counts using a triaxial accelerometer (Activestyle Pro HJA-750C; Omron Healthcare Co., Ltd., Kyoto, Japan).^4^ Participants were to attach the accelerometer to their waist each day, except while bathing and sleeping. We assessed step counts from the date the WHO declared the COVID-19 pandemic (March 12, Japan standard time) until the date the Japanese government lifted the state of emergency (May 25), omitting outliers (<100 or >20,000 steps/day). We averaged daily step counts every 7 days, to account for slight variations across days, and calculated the percent change in step counts every 7 days based on the average daily step counts during the 1 month before the WHO declaration as a reference period (ie, February 13 to March 11, Japan standard time).

**Figure 1.**
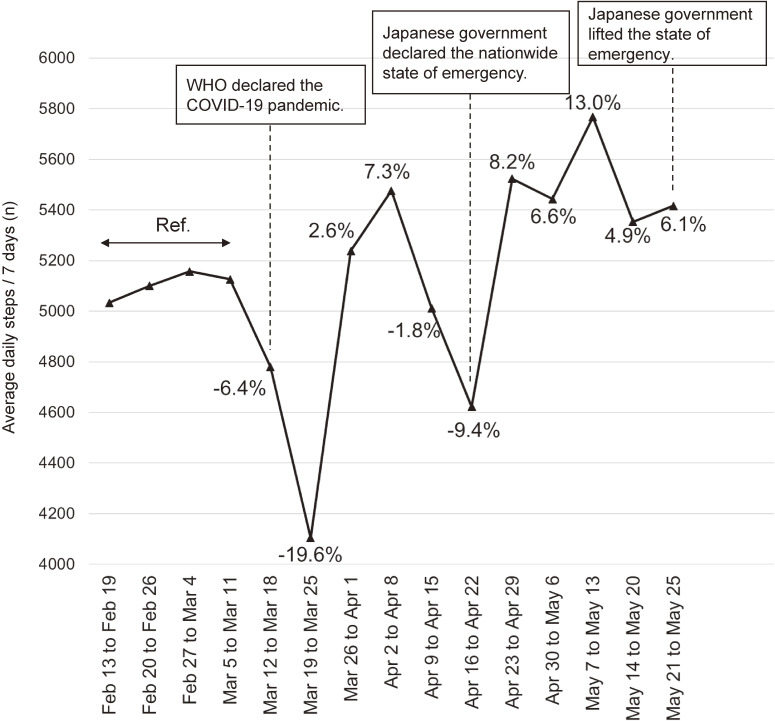
Average daily steps/7 days during COVID-19 pandemic and percent change from before March 12, 2020: The Masuda Study. Step counts were analyzed from the date the COVID-19 pandemic was declared by the WHO (March 12, 2020, Japan standard time) until the date the state of emergency in Japan was lifted by the Japanese government (May 25, 2020). We averaged daily step counts every 7 days and calculated the percent change in step counts every 7 days, based on the average daily step counts during the 1 month prior to the WHO declaration as a reference period (ie, February 13 to March 11, 2020, Japan standard time). COVID-19, coronavirus disease 2019; WHO, World Health Organization.

We observed a total of 11,927 daily step count measurements over the study period. Within 1 week of the declaration, mean daily step counts decreased by 6.4% (by 325 steps); by the following week, step counts had decreased by 19.6% (by 1,000 steps) compared with step counts before the declaration. Two weeks after the WHO declaration, step counts recovered to nearly reference levels (Figure 1). Within a week of nationwide state of emergency initiation in Japan, mean daily step counts decreased by 9.4% (by 482 steps); however, step counts had recovered the following week. There were no remarkable decreases in mean daily step counts, thereafter, until the state of emergency was lifted.

Despite the non-enforceable restrictions in Japan during the COVID-19 pandemic, transient rapid declines occurred in physical activity among participants, measured using daily step counts, which may reflect social distancing measures and adherence to social distancing. Relatively rapid recovery in step counts by 2 weeks after the declarations may be because the Japanese government could not legally enforce lockdowns on citizens and residents; these strategies relied largely on voluntary self-restriction. Another explanation may be that there were no COVID-19 infections reported in Masuda city during the study period.^5^ Some study limitations warrant consideration. The study sample was small and obtained from a single rural area of Japan, which may limit generalizability of our findings. Second, there may be a systematic reporting bias owing to changes in participants’ behavior when wearing the device or non-compliance with using the device. Third, we did not assess activity intensity or non-stepping exercise, such as cycling. Further studies with a large sample size across Japan are required to confirm our findings. The influence on overall physical activity owing to social distancing measures, particularly if prolonged, is important to consider.
